# The relationship between single and two-dimensional indices of left ventricular size using hemodynamic transesophageal echocardiography in trauma and burn patients

**DOI:** 10.1186/s13089-017-0074-z

**Published:** 2017-10-11

**Authors:** Duraid Younan, T. Mark Beasley, David C. Pigott, C. Blayke Gibson, John P. Gullett, Jeffrey Richey, Jean-Francois Pittet, Ahmed Zaky

**Affiliations:** 1 Department of Surgery, Division of Acute Care Surgery, University of Alabama at Birmingham, Birmingham, AL USA; 2 Department of Biostatistics, University of Alabama at Birmingham, Birmingham, AL USA; 3 Department of Emergency Medicine, University of Alabama at Birmingham, Birmingham, AL USA; 4 Department of Anesthesiology and Perioperative Medicine, University of Alabama at Birmingham, Birmingham, AL USA; 5 Birmingham/Atlanta VA Geriatric Research, Education, & Clinical Center, Department of Veteran’s Affairs, Birmingham, AL USA

**Keywords:** Transesophageal echocardiography, Left ventricular area, Left ventricular diameter, Resuscitation, Critical care, Ultrasonography, Trauma

## Abstract

**Background:**

Conventional echocardiographic technique for assessment of volume status and cardiac contractility utilizes left ventricular end-diastolic area (LVEDA) and fractional area of change (FAC), respectively. Our goal was to find a technically reliable yet faster technique to evaluate volume status and contractility by measuring left ventricular end-diastolic diameter (LVEDD) and fractional shortening (FS) in a cohort of mechanically ventilated trauma and burn patients using hemodynamic transesophageal echocardiographic (hTEE) monitoring.

**Methods:**

Retrospective chart review performed at trauma/burn intensive care unit (TBICU). Data on 88 mechanically ventilated surgical intensive care patients cared for between July 2013 and July 2015 were reviewed. Initial measurements of LVEDA, left ventricular end-systolic area (LVESA) and FAC were collected. Post-processing left ventricular end-systolic (LVESD) and end-diastolic diameters (LVEDD) were measured and fractional shortening (FS) was calculated. Two orthogonal measurements of LV diameter were obtained in transverse (Tr) and posteroanterior (PA) orientation.

**Results:**

There was a significant correlation between transverse and posteroanterior left ventricular diameter measurements in both systole and diastole. In systole, r = 0.92, p < 0.01 for LVESD-Tr (mean 23.47 mm, SD ± 6.77) and LVESD-PA (mean 24.84 mm, SD = 8.23). In diastole, *r* = 0.80, *p* < 0.01 for LVEDD-Tr (mean 37.60 mm, SD ± 6.45), and LVEDD-PA diameters (mean 42.24 mm, SD ± 7.97). Left ventricular area (LVEDA) also significantly correlated with left ventricular diameter LVEDD-Tr (*r* = 0.84, *p* < 0.01) and LVEDD-PA (*r* = 0.90, *p* < 0.01). Both transverse and PA measurements of fractional shortening were significantly (*p* < 0.0001) and similarly correlated with systolic function as measured by FAC. Bland–Altman analyses also indicated that the assessment of fractional shortening using left ventricular posteroanterior diameter measurement shows agreement with FAC.

**Conclusions:**

Left ventricular diameter measurements are a reliable and technically feasible alternative to left ventricular area measurements in the assessment of cardiac filling and systolic function.

## Background

Historically, the assessment of volume status and cardiac function with the goal of achieving appropriate resuscitation targets has been an area of ongoing interest to intensivists worldwide. The pulmonary artery catheter has long been used in the intensive care unit (ICU) to evaluate volume status, cardiac function, and to guide resuscitation. Recently, multiple studies have questioned the benefits of pulmonary artery catheter use and increased the awareness of associated complications, resulting in a decline in its use [1, 2].

Recently, the debate between static and dynamic indices of volume responsiveness was resolved in favor of the latter with multiple studies demonstrating that central venous pressure lacks predictability as a measure of volume responsiveness [3] compared with stroke volume and pulse pressure variation [4]. It is important to mention, however, that the validity of dynamic indices is limited by the presence of spontaneous respiration, dysrhythmia, or vasopressor use [5].

Over the last few years, the use of bedside ultrasonography and echocardiography has expanded both in trauma and ICU settings; surgeons, intensivists, and emergency care physicians have developed training protocols to facilitate the ability to detect free fluid in the abdomen with high sensitivity and specificity [6]. The BEAT exam (Bedside Echocardiographic Assessment in Trauma/Critical Care) is an example of a bedside hemodynamic evaluation protocol that has been developed to assess stroke volume, the presence of pericardial or pleural effusion, ventricular function, size, and volume status [7].

Monoplane hemodynamic transesophageal echocardiography (hTEE; ImaCor, Inc., Garden City, NY) is a relatively new diagnostic tool, allowing the intensivist to directly assess both the contractility and filling status of both the right and left ventricles at the bedside in real-time. Unlike conventional transesophageal echocardiography (TEE) probes, the hTEE probe is smaller (5.5 mm in diameter), disposable, and can remain in place for up to 72 h, permitting continuous visual quantitative estimation of cardiac contractility and cardiac filling. The probe can be placed safely [8] in intubated patients by intensivists with a basic level of hTEE training without the need for formal training in conventional TEE [9]. Additionally, hTEE is only capable of displaying three echocardiographic windows compared with 28 views in the case of conventional TEE. The diagnostic yield of hTEE was shown to be non-inferior to thermodilution in the postoperative care of cardiac surgical patients [10]. In fact, information recovered from hTEE led to changes in the plan of care for those patients compared to those evaluated solely with thermodilution [8, 10]. In addition, hTEE was proven useful in weaning from ventriculoarterial extracorporeal membrane oxygenation (VA ECMO) [11], and has led to changes in intensive care management in patients with left ventricular assist devices [12]. To date, however, no validation studies have been performed to compare hTEE with conventional multiplane TEE.

Echocardiographically estimated fractional area of change (FAC) calculated as the percentage change between left ventricular end-systolic and end-diastolic areas (LVESA and LVEDA, respectively) has been used as a surrogate marker for left ventricular ejection fraction (and thus, systolic function). Similarly, LV preload can also be assessed using LVEDA with consistent accuracy [13]. Despite their obvious appeal, LV area-based measurements are time-consuming and challenging to obtain due to technical issues associated with chamber border detection. These measurements become even more challenging during periods of hemodynamic instability. Additionally, hTEE is incapable of measuring ejection fraction (EF) because it lacks the echocardiographic windows necessary to measure EF such as the midesophageal 2-chamber view. This limitation becomes more evident in current-generation hTEE systems that lack software capable of calculating end-systolic and end-diastolic volumes and hence performing automatic ejection fraction calculation. The current hTEE systems are only capable of calculating end-diastolic and end-systolic areas, requiring multiple manual steps for LV FAC calculation compared with conventional TEE systems that are capable of volumetric EF calculation.

In this study, the authors used hTEE to obtain LV diameter measurements to estimate left ventricular systolic function (using left ventricular fractional shortening), and filling status (using left ventricular end-diastolic diameter) as a technically feasible, less time-consuming alternative to area-based measurements.

## Methods

This is a retrospective chart review performed at the trauma and burn intensive care unit (TBICU) at the University of Alabama at Birmingham. After Institutional Review Board (IRB) approval, medical records and hTEE video clips of patients admitted to our TBICU between July 1, 2013 and July 1, 2015 were reviewed. Inclusion criteria were all mechanically ventilated trauma and burn patients admitted to our TBICU during the study period who underwent hTEE exam to evaluate volume status and hemodynamic stability, or with persistent hypotension (systolic blood pressure < 90 mmHg after a 1 L fluid bolus). Patients with poor-quality echocardiographic windows or with known or recognized wall motion abnormalities as agreed upon by two hTEE-trained examiners (DY and JR) or with incomplete data were excluded from the study.

hTEE exams were performed by an intensivist, resident staff, or a trained mid-level provider (physician assistant). We used all echocardiographic views available by hTEE to perform our evaluation of cardiac function and filling. We did not endorse a specific protocol for resuscitation. Personnel who performed the hTEE measurements were all previously trained in performing focused transthoracic echocardiography (TTE). Personnel who performed the hTEE measurements also attended a dedicated 6-hour hTEE training session. A total of ten hTEE exams performed independently were deemed sufficient to achieve competence. Exams were performed using monoplane hTEE (ImaCor ClariTEE, ImaCor, Inc., Garden City, NY). hTEE cineloops were stored and analyzed on the Imacor Zura imaging system (Imacor, Inc., Garden City, NY). The hTEE system used in this study was not equipped with automated border detection technology.

**Fig. 1:**
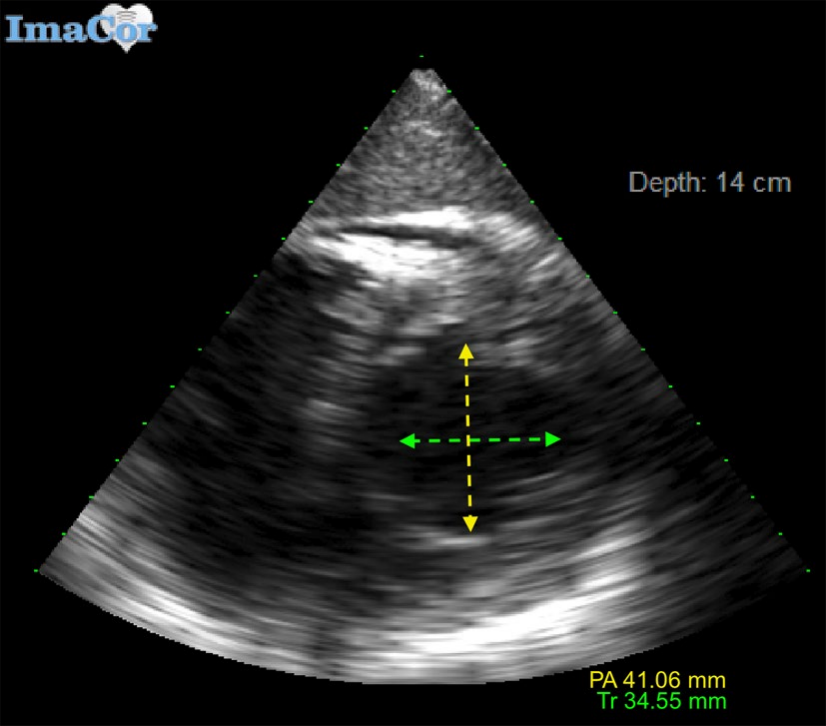
Transesophageal echocardiographic view demonstrating transverse and posteroanterior measurements of left ventricle in end-diastole

**Fig. 2:**
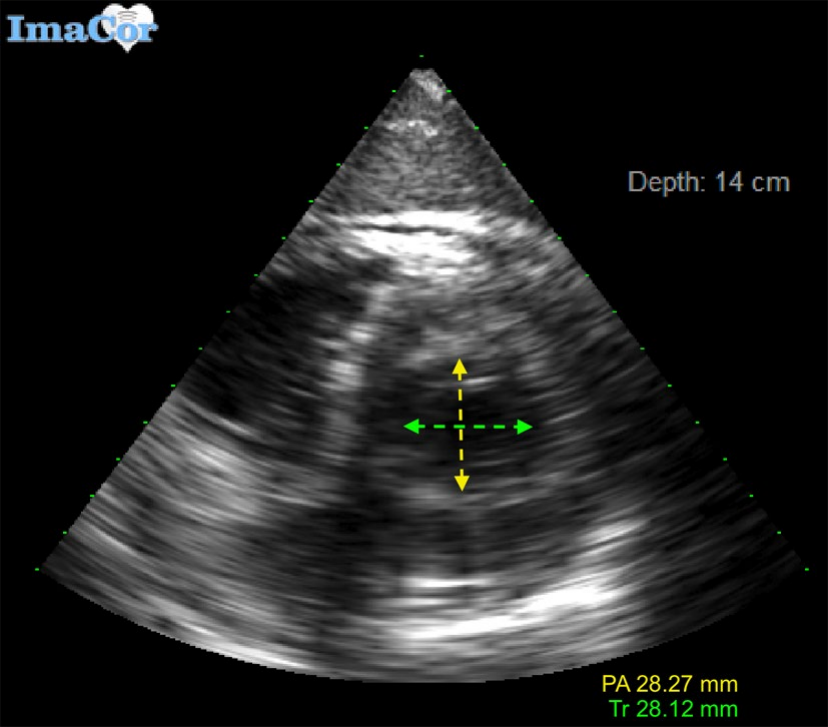
Transesophageal echocardiographic view demonstrating transverse and posteroanterior measurements of left ventricle in end-systole

We obtained two perpendicular LV diameter measurements from video clips of the standard transgastric mid-papillary short-axis view. Diameter 1 (transverse–Tr) was obtained by measuring between the papillary muscles from the lateral wall to the septal wall in a transverse orientation (i.e., from 3 to 9 o’clock) while Diameter 2 (postero-anterior–PA) was obtained by measuring from the inferior wall to the anterior wall (corresponding to an inner diameter drawn between the mid-inferior and mid-anterior segments in the standard 17-segment echocardiographic model) [14], medial to the papillary muscles in a top-to-bottom orientation (i.e., from 12 to 6 o’clock) (Figs. 1, 2).

**Fig. 3:**
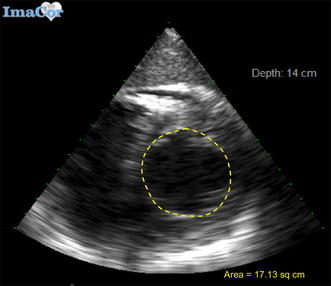
Transesophageal echocardiographic view demonstrating area tracing of left ventricle in end-diastole

**Fig. 4:**
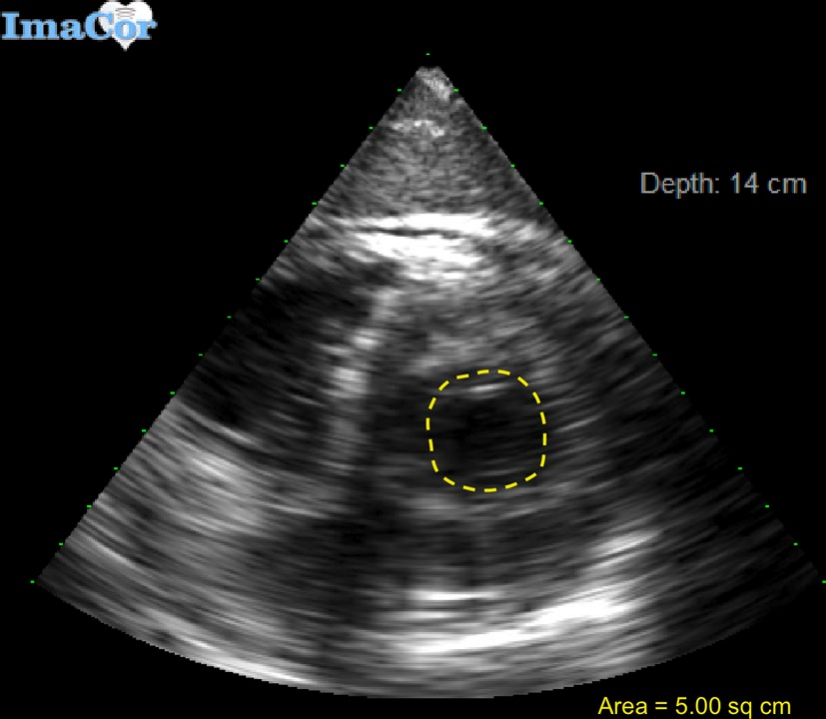
Transesophageal echocardiographic view demonstrating area tracing of left ventricle in end-systole

Maximum end-diastolic diameters were determined by electrocardiography and echocardiography. All measurements were agreed upon by two hTEE-trained examiners (DY and JR). Evaluation of cardiac filling and contractility were obtained by using LVEDA and LVESA (Figs. 3, 4) measurements to calculate LV FAC, according to the following formula: FAC = (LVEDA − LVESA)/LVEDA. Transesophageal four-chamber views were not utilized in this study.

Left ventricular endocardial fractional shortening (FS), a surrogate marker for LV systolic function, was calculated using the formula, FS = (LVEDD − LVESD)/LVEDD, corresponding to the FAC formula based on LVEDA and LVESA measurements.

### Statistical analysis

Pearson correlations were used to assess the relationships between area- and diameter-based measurements and FAC and FS ratios. With a sample size of approximately 100, we had 80% statistical power to detect correlation coefficients of *r* = 0.277 (*r* ^2^ = 0.073) or larger at a 5% level. The relationship between area- and diameter-based measurements and FAC were also examined using the Bland–Altman technique, which compares the difference in two measures to their mean. Specifically, the bias in measurement is reflected in the mean difference in measures, which was tested with a paired *t* test. Given the strong correlations among these measures, we had 80% statistical power to detect a difference of 0.16 standard deviation units at a 5% significance level. The covariance (correlation) between the difference in the mean is used to assess agreement, in that a significant correlation indicates poor agreement. The limits of agreement are defined as bias ± 1.96 (standard deviation of bias). All statistical analyses were performed using SAS 9.3 (SAS Institute, Inc., Cary, NC).

## Results

A total of 107 echocardiographic studies were performed during the study period. Eight patients had incomplete data and were excluded from the study, resulting in a total of 88 patients with 99 measurements.

Table 1 shows patient demographics. Patients were predominantly white, male with a mean Injury Severity Score (ISS) of 23.4 ± 12.1. The mean LVEDD-Tr was 37.60 mm (SD ± 6.45), whereas the mean LVEDD-PA was 42.24 mm (SD ± 7.97). A paired *t* test indicated that this difference in means was significant (*p* < 0.0001). There was a significant correlation between LVEDD-Tr and LVEDD-PA diameters (*r* = 0.80, *p* < 0.0001). The intraclass correlation coefficient (ICC) for LVEDD measures was 0.83.

**Table 1:** Patient demographics, *N* = 88

	(M ± SD)
Age (years)	49.7 ± 20.7
IVF (liters)	6.7 ± 5.5
Lactic acid (mg/dL)	2.1 ± 1.6
HCT (%)	27.0 ± 5.1
Time from admission to procedure (days)	7.3 ± 8.5
ICU stay (days)	26.8 ± 19.1
ISS	23.4 ± 12.1
Race	*N* (%)
White	53 (60.2%)
Black	29 (33.0%)
Other	7 (6.8%)
Gender *N* (%)	
Male	68 (77.3%)
Female	20 (23.7%)
Injury type *N* (%)	
Trauma-blunt	50 (56.8%)
Trauma-penetrating	14 (15.9%)
Burn	24 (27.3%)
Indication for test *N* (%)	
Sepsis	26 (29.9%)
Trauma/burn volume assessment	56 (63.3%)
Spinal cord injury (SCI)	1 (1.1%)
ARDS	4 (4.6%)
Cardiogenic shock	1 (1.1%)

The mean LVESD-Tr was 23.47 mm (SD ± 6.77); the mean LVESD-PA was 24.84 mm (SD ± 8.23); however, a paired *t* test indicated that this difference in means was significant (t (98) = 4.10, *p* < 0.0001). Pearson correlation indicated that the transverse and PA LVESD measures were significantly correlated (*r* = 0.92, *p* < 0.0001). The ICC for these LVESD measures was 0.93.

We also compared area-based (LVEDA) with diameter-based measurements (both transverse and PA LVEDD) as surrogates for LV preload. The LVEDA significantly correlated with LVEDD-Tr (*r* = 0.84, *p* < 0.0001) and LVEDD-PA (*r* = 0.90, *p* < 0.0001). Similarly, LVESA significantly correlated with LVESD-Tr (*r* = 0.90, *p* < 0.0001) and LVESD-PA (*r* = 0.92, *p* < 0.0001), respectively, suggesting that diameter-based and area-based measurements provide a similar assessment of LV preload.

We compared area-based FAC with diameter-based FS in both PA and transverse planes to evaluate contractility. Table 2 shows that both transverse- and PA-measured FS are significantly and similarly correlated to FAC (*p* < 0.0001), indicating that each of these measurements has the potential to serve as proxy measure for contractility. The Bland–Altman technique tests the relationship between the mean of the two measures (e.g., FS and FAC) and their difference. A statistically significant relationship between the mean and the difference indicates that the two measures are not in close agreement. The results in Table 2 indicate a lack of agreement between FS-Tr and FAC. FS-PA, however, does show reasonable agreement with FAC, suggesting that it may be a feasible technique for estimating LV systolic function. There were no complications of probe placement in any of the patients included in this study.

**Table 2:** Mean ± standard deviations for FAC, FS and Pearson correlations for FAC with FS (both transverse and PA)

*N* = 99	M ± SD	Correlation with FAC	Bland–Altman correlation
FAC	58.08 ± 16.45		
FS transverse	37.74 ± 13.29	*r* = 0.85 *p* < 0.0001	*r* = 0.38 *p* = 0.0001
FS PA	41.16 ± 15.29	*r* = 0.85 *p* < 0.0001	*r* = 0.14 *p* = 0.1707

FAC = (LVEDA − LVESA)/LVEDA; FS = (LVEDD − LVESD)/LVEDD

*p* values for all correlations are *p* < 0.0001

## Discussion

This study demonstrates a correlation between area-based and diameter-based measurements of left ventricular contractility and preload. Despite this correlation, hTEE-based posterior–anterior and transverse measurements used in diameter-based calculations significantly differed in a cohort of mechanically ventilated trauma and burn patients. This difference may be explained by ventricular shape and geometry.

Our study is in line with previous studies that have used end-diastolic diameter as a surrogate marker for preload and in the assessment of contractility. Inoue et al. [15] in a cohort of 166 patients on hemodialysis found LVEDD to be an independent predictor of mortality. Our study differed from Inoue in that it was conducted on surgical patients and was not designed to study outcomes. Indovina et al. [16]. found that LVEDD better discriminates between patients with depressed and normal LV systolic function (based on ejection fraction) compared with LV end-diastolic volume measured by blood pool-gated radioisotopic angiography. The authors in that study used the end-diastolic transverse diameter. LVEDD was also found to be an independent predictor of recovery of left ventricular function after therapy in patients with dilated cardiomyopathy [17]. From the above-mentioned studies, it is reasonable to conclude that LVEDD has utility in the assessment of LV preload and contractility.

In our study, both transverse and PA FS measurements correlated with area-based FAC measurements. The higher correlation of transverse diameter compared with PA diameter with FAC measurements can likely be explained by the relatively easier and hence more consistent measurements of LV end-diastolic transverse diameter compared with LV end-diastolic PA diameter. It is important to note that both PA and transverse diameters are linear measurements and may vary from area measurement based on LV geometry in health and in disease. Equally important, LV geometry should only be measured in the context of accurate and adequate image acquisition, since imaging errors such as foreshortening may be misinterpreted as a change in LV geometry.

Additionally, diameter-based measurements, due to their linear nature, may not be applicable for patients with regional wall motion abnormalities such as the elderly or those with known ischemic heart disease. It is also important to note that the use of either area or diameter measurement does not preclude other methods of assessment and that the purpose of this study is to highlight a more feasible way of assessing cardiac filling and contractility for clinicians who are not formally trained in echocardiography. Whether a difference in clinical outcomes can be detected by either measurement remains to be determined in future studies.

As of now, there is no consensus on a formal training protocol for the use of hTEE as compared with conventional TEE. As a result, trainees are exposed most commonly to training protocols performed by the company representatives or by a trained echocardiographer at the bedside. Although hTEE training protocols provide significant utility, they may vary between institutions and currently lack accreditation. Likewise, hTEE-guided resuscitation protocols also vary between institutions and have not yet been validated in multicenter trials. A consensus on the appropriate level of mastery for basic hTEE use and goal-directed, hTEE-guided therapies is an important topic for future exploration in adequately powered studies.

## Limitations

Our study suffers from several limitations. First, diameters are linear rather than volume measurements. As such, they are more prone to missing LV pathology not incorporated in the area of measurement. It should be noted, however, that our study demonstrated a significant agreement between LV diameter and area. Second, an LV with greater mass may require higher filling pressures to manifest a change in diameter compared with an LV with smaller mass. Finally, our study suffers from the limitations of retrospective studies such as missing data, lack of a gold standard against which our measurements were made, reliance on qualitative rather than quantitative agreements between hTEE-trained individuals, and the absence of concomitant conventional transthoracic echocardiography to rule out cardiac abnormalities undetected by hTEE. However, we would like to emphasize that the goal of this paper was to test the correlation between two TEE-based methods of assessing cardiac filling and contractility. We did not attempt to explore the adequacy of either measurement in terms of outcomes or in terms of comparison to a gold standard.

## Conclusions

Further prospective, adequately powered studies are required to compare diameter-based measurements with previously validated volumetric measurements of left ventricular preload and contractility using a more standardized level of operator training. Similarly, the utility of diameter-based echocardiographic assessment for the evaluation of volume responsiveness and to guide resuscitation must be validated before its role in the resuscitation of critically ill or injured patients can be clearly established.

In conclusion, left ventricular diameter-based measurements are reliable, less technically challenging alternatives to area-based measurements in the assessment of cardiac filling and LV contractility. More studies are needed to compare the use of diameter-based measurements to left ventricular volumes as a gold standard for assessing left ventricular systolic function and preload. More studies are needed to examine the feasibility of performing LVEDD by less-experienced caregivers.

## Abbreviations

LV: left ventricle
LVEDA: left ventricular end-diastolic area
FAC: fractional area of change
LVEDD: left ventricular end-diastolic diameter
FS: fractional shortening
LVEDS: left ventricular end-systolic diameter
hTEE: hemodynamic transesophageal echocardiography
LVESA: left ventricular end-systolic area
LVEDD-Tr: left ventricular end-diastolic diameter, transverse
LVEDD-PA: left ventricular end-diastolic diameter, posteroanterior
ICU: intensive care unit
BEAT: Bedside Echocardiographic Assessment in Trauma/Critical Care
TEE: transesophageal echocardiography
ICC: intraclass correlation coefficient
